# Lethal, Sublethal, and Offspring Effects of Fluralaner and Dinotefuran on Three Species of *Bactrocera* Fruit Flies

**DOI:** 10.3390/insects15060440

**Published:** 2024-06-11

**Authors:** Doudou Li, Xinyan Cai, Yixiang Qi, Yongyue Lu, Xinlian Li

**Affiliations:** Department of Entomology, College of Plant Protection, South China Agricultural University, Guangzhou 510640, China; lddoui@163.com (D.L.); xinyan_cai@126.com (X.C.); qiyixiang19880922@163.com (Y.Q.)

**Keywords:** ecotoxicology, insecticide-resistance management (IRM), invasive pests, pesticide, reproduction, survival

## Abstract

**Simple Summary:**

*Bactrocera dorsalis*, *Bactrocera cucurbitae*, and *Bactrocera tau* are three invasive fruit fly pests that can cause significant economic damage to crops. Chemical agents have become the primary method for improving fruit fly management, leading to the development of insecticide resistance in these three species. Here, we assess the toxicity of a novel insecticide (fluralaner) and a commonly used insecticide (dinotefuran) against these three fruit fly species. Both insecticides exhibit strong lethal and sublethal effects on adult fruit flies and on the sex ratio of their offspring. Hence, fluralaner and dinotefuran can significantly control *Bactrocera dorsalis*, *B. cucurbitae*, and *B. tau*. These findings can help optimize insecticide application and ensure effective management of insecticide resistance.

**Abstract:**

Fruit flies cause substantial economic damage, and their management relies primarily on chemical insecticides. However, pesticide resistance has been reported in several fruit fly species, the mitigation of which is crucial to enhancing fruit fly control. Here, we assess the toxicity of a novel insecticide (fluralaner) and a common insecticide (dinotefuran) against three fruit fly species, *Bactrocera dorsalis* (Hendel), *Bactrocera cucurbitae* (Coquillett), and *Bactrocera tau* (Walker). Both pesticides exhibit robust lethal and sublethal effects against all three fruit fly species, with fluralaner being more potent. Fluralaner and dinotefuran suppress the reproductive capacities and survival rates of fruit flies. However, at the 50% lethal concentration, fluralaner stimulates the reproductive capacity of *B. dorsalis* and the survival rate of *B. tau*. Fluralaner also causes significant transgenerational effects, impacting the offspring hatching rate of *B. cucurbitae* and *B. tau* and reducing the proportion of female offspring. Thus, both pesticides exhibit high potential for controlling fruit flies. However, their application should be tailored according to species variations and the diverse effects they may induce. Collectively, the findings of this study outline the sublethal effects of two insecticides against fruit flies, helping to optimize their application to ensure the effective management of insecticide resistance.

## 1. Introduction

*Bactrocera dorsalis* (Hendel), *Bactrocera cucurbitae* (Coquillett), and *Bactrocera tau* (Walker) are important invasive fruit fly pests of international concern that can cause significant economic crop damage [1,2,3,4,5]. In China, these three species are widely distributed in regions like Taiwan, Fujian, and Guangdong, causing annual outbreaks and proving difficult to control. In fact, they have exhibited trends of a gradual spread northward [6,7,8]. Ongoing research and scientific advancements have yielded diverse methods for the management of fruit flies, including RNA interference pesticides, insect sterilization techniques, and various trapping methods. However, chemicals remain the most commonly used modality of control [9,10].

Fluralaner is a representative isoxazoline broad-spectrum insecticide that inhibits the γ-aminobutyric acid-gated chloride channels of pests, representing a novel mode of action with no significant cross-resistance to other insecticides [11,12,13]. Fluralaner exhibits excellent insecticidal activity against over 30 insect species [14] while proving safe for non-target organisms (e.g., mammals, zebrafish, and poultry), suggesting broad application prospects [15,16,17]. Meanwhile, dinotefuran is a third-generation neonicotinoid insecticide that exerts insecticidal effects primarily by acting on nicotinic acetylcholine receptors in insects [18,19,20]. Although the use of neonicotinoid insecticides in agricultural and domestic applications is rapidly growing [21], their application in managing fruit flies has not been adequately investigated.

In practical applications, insecticides may directly induce lethal and sublethal effects. The latter can influence the behavior, physiological activities, or survival capabilities of insects over a particular period [22,23]. For instance, flupyradifurone—a butanolide insecticide that targets the central nervous system of insects in a manner similar to neonicotinoid—prolongs the development of *Binodoxys communis* Gahan while reducing its longevity [24,25]. Additionally, the sublethal effects of the neonicotinoid insecticide thiacloprid may affect the learning and memory behaviors of *Apis mellifera* L. [26]. Meanwhile, lethal and sublethal concentrations of cyantraniliprole (bisamide) affect the mating performance of *B. dorsalis* adults [27], whereas broflanilide—a meta-diamide insecticide—at its lethal concentration 50 (LC_50_) affects the hatchability of *B. cucurbitae* and *B. tau* [3].

This study tests the hypothesis that fluralaner and dinotefuran exhibit insecticidal activity against *B. dorsalis*, *B. cucurbitae*, and *B. tau*. More specifically, their lethal and sublethal effects on fruit flies are evaluated, as well as their transgenerational effects. The results of this study provide a theoretical foundation for the future application of fluralaner and dinotefuran in controlling fruit flies in the field.

## 2. Materials and Methods

### 2.1. Insect Strains and Insecticides

The three fruit fly species used in this study (*B. dorsalis*, *B. cucurbitae*, and *B. tau*) were susceptible strains that had been cultured in a laboratory for over 60 generations [3]. The larvae and adults were reared on artificial diets [28,29]. Fluralaner (purity ≥ 99%, CAS: 864731-61-3) and dinotefuran (purity ≥ 99%, CAS: 165252-70-0) were purchased from Shanghai Yuanye Bio-Technology Co., Ltd. (Shanghai, China).

### 2.2. Determining the Lethal Concentrations of Fluralaner and Dinotefuran

The lethality of fluralaner and dinotefuran was determined using the residual contact method [28]. The chemicals were prepared in acetone as 100 mg/L stock solutions. Fluralaner and dinotefuran stock solutions were diluted to 5–6 different concentrations with acetone; 5 mL of each diluted solution was added to a separate 250 mL clean conical flask, which was gently rotated so that the solution evenly coated the inside of the flask until the acetone evaporated. Twenty adult flies (1:1 sex ratio) (3–5 days after emergence) were placed in a prepared conical flask, which was sealed with gauze that had cotton dipped in 10% honey water placed on top (three replicates per concentration). The mortality rate was observed after 24 h. The adults were gently turned over with a brush and considered dead if they could not turn back over within 30 s. If the mortality rate in the control group (acetone only) was <10%, the experiments were considered valid, and the adjusted mortality was corrected using Abbott’s formula [30]; if the mortality rate in the control group exceeded 10%, the experiment was considered invalid and repeated.

### 2.3. Bioassays

The lethal and sublethal effects of fluralaner and dinotefuran were studied under four separate conditions: treatment with either insecticide at the 15% lethal concentration (LC_15_), LC_30_, LC_50_, and a vehicle control with acetone. After exposure to different treatments for 24 h, we selected 20 pairs of healthy adult flies (5 days after emergence; F0 generation) and transferred them to breeding cages for rearing (*n* = 3 replicates/treatment). Please refer to Section 2.2 for treatment details. Subsequently, adult mortality and egg laying were observed daily, starting on the second day (6 days after emergence). After the adults began laying eggs, eggs were collected every 3 days for 5 h until all the *B. dorsalis* flies died (eggs were collected 50 times each for *B. cucurbitae* and *B. tau*). Due to differences in the oviposition habits between the fruit fly species, oviposition cups with orange juice were used to collect *B. dorsalis* or eggs, and with pumpkin flesh to collect *B. cucurbitae* and *B. tau* eggs; simultaneously, the oviposition quantity of each species was recorded. Additionally, the egg-hatching rate, female fecundity, survival rate (male and female), and F1 sex ratio (proportion of females  =  Σ♀  ÷  Σ (♀  +  ♂)) were observed after each treatment to assess the sublethal effects of both agents on all three species of fruit flies [3]. Population parameters were calculated according to the protocol described by Zhang et al. [31].

### 2.4. Data Analysis

Probabilistic regression analyses were performed using SPSS version 22.0 (SPSS, Inc., IBM, Armonk, NY, USA) to calculate the LC_50_, LC_30_, and LC_15_ values of both pesticides against fruit flies and the corresponding 95% confidence limits (CLs). The SAS software (version 9.4) was used for all other data analyses. Significance analyses of the experimental results were performed using Duncan’s multiple-range test (DMRT) or the *t*-test (with validation of normal distribution before data analysis). We set *p* < 0.05 as the threshold for statistical significance. The data generated in this study were subjected to analysis of variance (ANOVA) followed by Tukey’s or Friedman’s post hoc tests.

## 3. Results

### 3.1. Toxicity of Fluralaner and Dinotefuran in Laboratory Settings

The bioassays demonstrated high virulence levels for fluralaner and dinotefuran in the adults of all three fruit fly species. Notably, the LC_50_ of fluralaner was consistently lower than that of dinotefuran in all the species (Table 1). The LC_50_, LC_30_, and LC_15_ values of both pesticides against the various fruit fly species were calculated for the subsequent experiments.

### 3.2. Sublethal Effects on Survival and Reproduction

**Figure 1:**
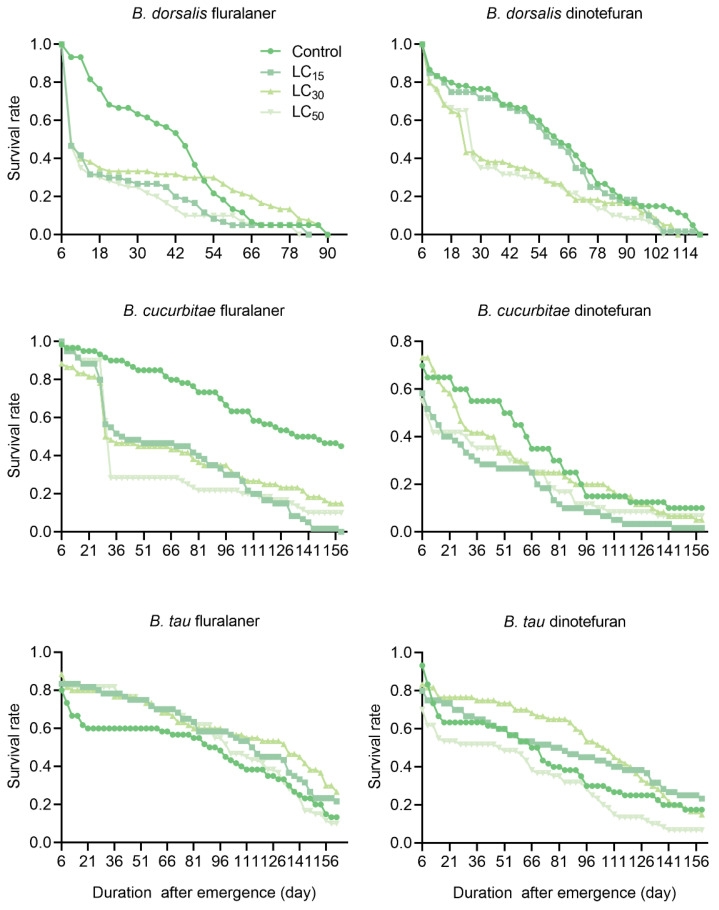
Effects of fluralaner and dinotefuran treatments on the survival of female *Bactrocera* fruit flies.

**Figure 2:**
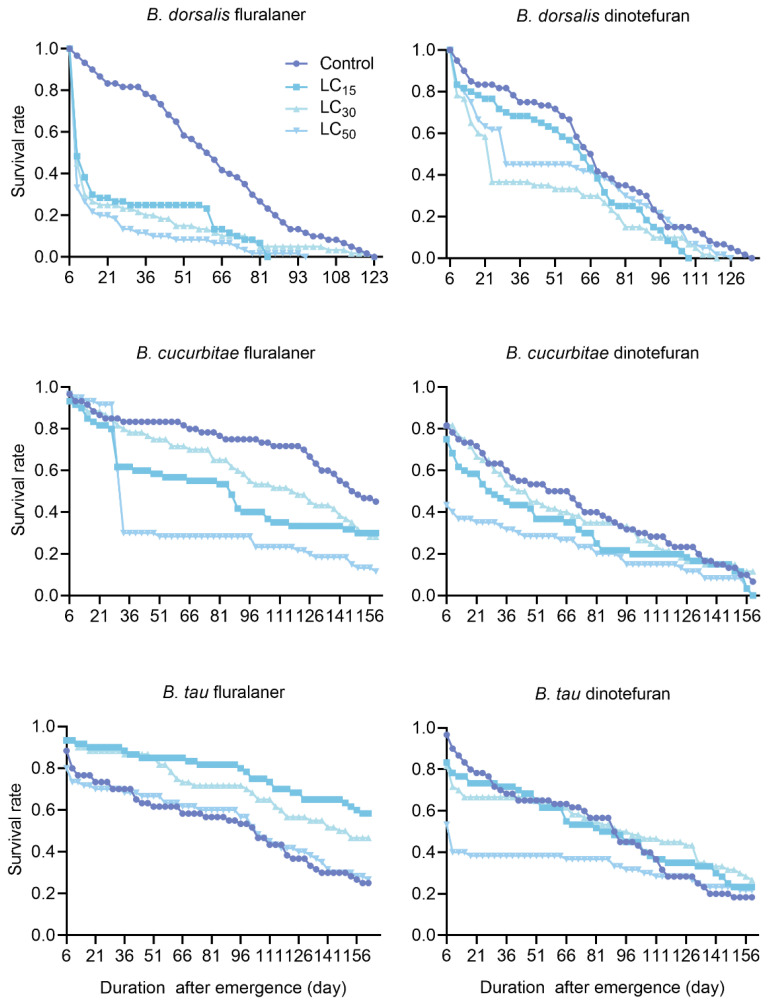
Effects of fluralaner and dinotefuran treatments on the survival of male *Bactrocera* fruit flies.

*Bactrocera dorsalis* survival decreased significantly after exposure to fluralaner or dinotefuran. However, the survival of the males was significantly lower than that of the females following fluralaner treatment (LC_30_ and LC_50_), whereas no difference was observed between the males and females treated with dinotefuran (Table 2). Almost half of both the male and female *B. dorsalis* flies died on day 4 (emergence at day 5 was considered the first day of treatment) of fluralaner treatment (9 days after emergence), whereas dinotefuran (LC_30_ and LC_50_) showed a sharp decline in survival after 16 days of treatment (21 days after emergence; Figure 1 and Figure 2).

The treatment with fluralaner significantly reduced the survival rate of male and female *B. cucurbitae*, with a more pronounced effect observed in the females compared to the males (Table 2). With dinotefuran treatment, the survival rates of female and male *B. cucurbitae* flies did not differ from those of the control at LC_30_, although all other doses resulted in significantly lower survival than that of the control group. Unlike fluralaner treatment, the survival rates of both sexes under dinotefuran treatment were only significantly lower than that of the males at LC_15_ (Table 2). Male and female *B. cucurbitae* survival rates declined sharply after 22 days (27 days after emergence) of fluralaner treatment but declined relatively slowly after dinotefuran treatment. Nevertheless, male survival fell below 50% by the following day (6 days post-emergence) after dinotefuran treatment at LC_50_ (Figure 1 and Figure 2).

In contrast to *B. dorsalis* and *B. cucurbitae*, the male and female survival rates of *B. tau* were significantly higher in the fluralaner-treated groups than in the control group, with the females exhibiting significantly lower longevity than the males (LC_15_ and LC_30_). Meanwhile, dinotefuran treatment only reduced male and female survival significantly under LC_50_ treatment; males and females did not exhibit survival differences under any of the treatment doses (Table 2). The survival of both male and female *B. tau* exhibited a sharp decline on the second day (6 days post-emergence) of fluralaner or dinotefuran treatment; subsequently, their survival rates declined slowly (Figure 1 and Figure 2).

Neither pesticide altered the fecundity of *B. cucurbitae* or *B. tau*. However, both significantly altered the fecundity of *B. dorsalis* at their respective LC_30_ and LC_50_ values. Specifically, treating *B. dorsalis* with fluralaner at LC_50_ resulted in higher fecundity than that observed in the control group, whereas the remaining treatments resulted in significantly lower fecundity levels (Table 2).

### 3.3. Sublethal Effects on Offspring Traits

The effects of both pesticides on the hatchability of *B. dorsalis* eggs were negligible. For *B. cucurbitae*, only the LC_50_ dose of fluralaner significantly reduced egg hatchability, whereas for *B. tau*, both pesticides reduced egg hatchability (Table 2).

No differences were observed in the net reproductive rates (R_0_) of *B. cucurbitae* or *B. tau* between either of the insecticides and the control treatment. However, treating *B. dorsalis* with fluralaner at LC_15_, LC_30_, and LC_50_ reduced the R_0_ value, which was two-fold higher than the control treatment. In contrast, R_0_ exhibited a four-fold reduction after dinotefuran treatment at LC_30_ (Table 3). No significant differences were observed among any of the fruit flies, except with the dinotefuran treatment at LC_50_, which slightly reduced the interval between each *B. tau* generation. Only *B. dorsalis* showed significantly lower intrinsic and finite rates of increase upon fluralaner treatment (at LC_30_). In contrast, in *B. cucurbitae* and *B. tau*, these values did not differ significantly compared to the control group at any dose (Table 3).

**Figure 3:**
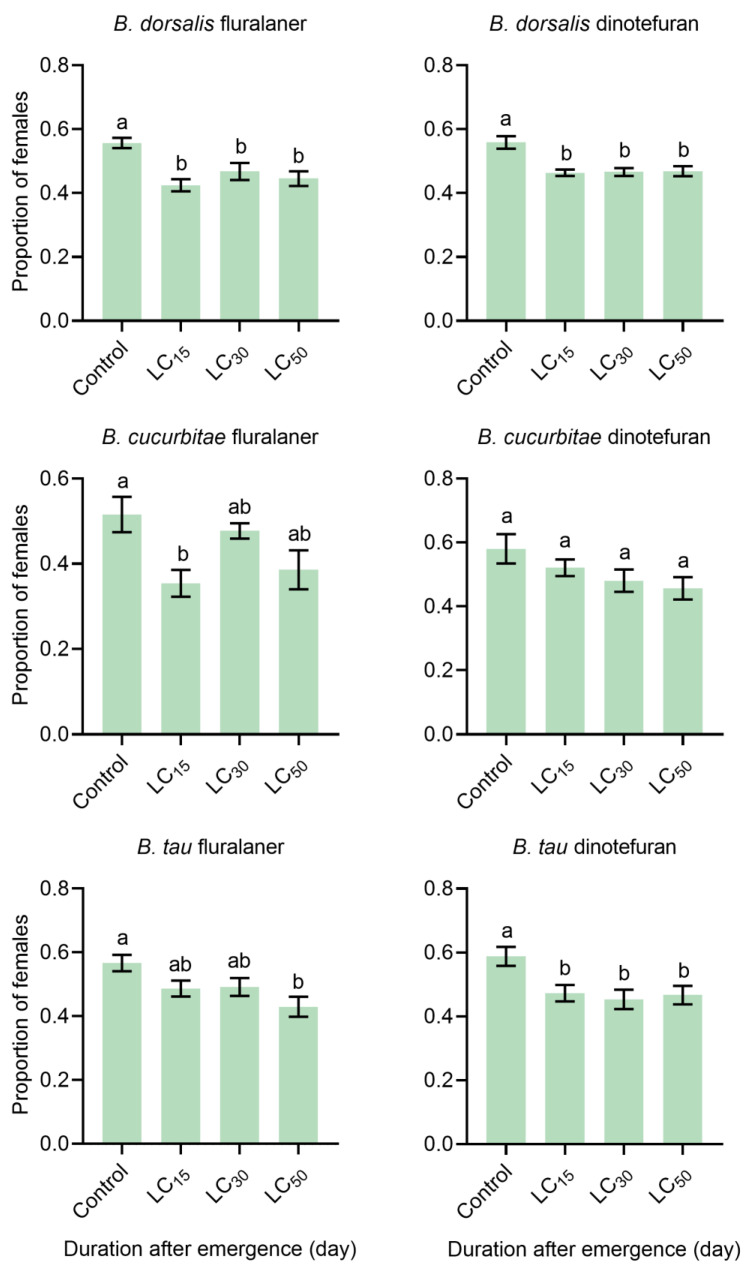
Effects of fluralaner and dinotefuran treatments on the sex ratios of fruit files: *Bactrocera dorsalis* treated with fluralaner (n = 22 replicates, F (3, 84) = 7.411, *p* = 0.0002), *B. dorsalis* treated with dinotefuran (n = 28 replicates, F (3, 108) = 9.785, *p* < 0.0001), *B. cucurbitae* treated with fluralaner (n = 12 replicates, F (3, 44) = 4.450, *p* = 0.0081), *B. cucurbitae* treated with dinotefuran (n = 13–17 replicates, F (3, 54) = 2.163, *p* = 0.1030), *B. tau* treated with fluralaner (n = 23 replicates, F (3, 88) = 4.127, *p* = 0.0087), and *B. tau* treated with dinotefuran (n = 18 replicates, F (3, 68) = 4.699, *p* = 0.0048). The data presented were analyzed by ANOVA followed by Tukey’s test or Friedman’s test. Different letters above the error bars indicate significant differences at the 0.05 level. The data in bar plots represent the mean ± the standard error of the mean (SEM).

Fluralaner and dinotefuran also affected the sex ratio of the offspring (Figure 3). Both pesticides decreased the proportion of female *B. dorsalis* progeny. This was also induced by dinotefuran treatment in *B. tau* and fluralaner treatment at the LC_50_ dose. In contrast, dinotefuran treatment did not alter the sex ratio of *B. cucurbitae* offspring, and only fluralaner at LC_15_ significantly reduced the proportion of female progeny.

## 4. Discussion

The management of fruit flies depends predominantly on the use of chemical insecticides. However, some fruit fly species, including *B. dorsalis* [32,33], *B. cucurbitae* [34], and *B. tau* [35,36] have developed resistance to several chemical agents. Hence, it is advisable to rotate the use of insecticides for effective insecticide resistance management (IRM). Fluralaner has recently emerged as a new agent with considerable effectiveness in treating ectoparasites [37]. Indeed, fluralaner exhibits high toxicity toward diverse agricultural insects [14]. Meanwhile, although dinotefuran is highly effective against various insects, it is rarely used for fruit fly control. Therefore, our research provides essential theoretical groundwork for the future use of fluralaner and dinotefuran in controlling fruit flies, as well as for improving IRM.

Our findings revealed that both fluralaner and dinotefuran were highly toxic to all three fruit fly species, with fluralaner being more effective. This phenomenon was also observed in *Plutella xylostella* (L.) larvae, with the LC_50_ of fluralaner being 0.02 mg/L, and that of dinotefuran exceeding 100 mg/L (feeding on a dipped leaf) [14,38]. The high toxicity of fluralaner has also been reported in *Sphodroxia maroccana* Ley [39] and *Spodoptera frugiperda* (J.E. Smith) [14], suggesting that fluralaner holds promise for diverse applications. Dinotefuran is widely used on a global scale; however, the associated resistance is steadily increasing in various insects, including *Nilaparvata lugens* (Stål) [40] and *Sogatella furcifera* (Horváth) [41]. Nevertheless, dinotefuran, which has not been extensively utilized for fruit fly control, may find potential application in the field through rotational strategies in the future.

The lethal or sublethal effects of insecticides [42] are achieved through their chemical components interfering with the insect’s physiological processes [42,43,44]. In this study, lethal and sublethal doses of both agents did not alter the fecundity of *B. cucurbitae* or *B. tau*. However, that of *B. dorsalis* significantly decreased under treatment with dinotefuran (LC_30_ and LC_50_) and fluralaner (LC_30_). Notably, treatment with fluralaner at LC_50_ significantly increased the reproductive capacity from 367.65 to 429.59 eggs/female. Indeed, the sublethal effects of insecticides can reportedly enhance or reduce the reproductive capacity of insects. For example, sublethal doses of sulfoxaflor decrease the reproductive capacity of *Coccinella septempunctata* L. [45], whereas *B. dorsalis*, *Bactrocera correcta* (Bezzi), and *B. cucurbitae* exhibit elevated fecundity after treatment with broflanilide [3]. Moreover, the sublethal effects of fluralaner and dinotefuran positively and negatively impacted the survival rates of males and females across all three fruit fly species, respectively.

Most sublethal effects tend to negatively impact the survival rates of insects, such as *Binodosys communis* [24,25]. In this study, we observed that the survival rates of the male and female *B. tau* insects increased after treatment with fluralaner (LC_15_ and LC_30_). Correspondingly, the hatching rate of the offspring decreased. Although the survival rate of the female *B. tau* insects (LC_30_) was also higher than that of the control group under dinotefuran treatment, no difference was observed between the males and the controls, resulting in no difference in the hatching rates. Collectively, these observations indicate that insects incur a fitness cost at sublethal insecticide concentrations [46,47]. This can cause insects to adopt complex survival strategies when facing chemical pressures in their environment, balancing survival, reproductive capacity, and the ability of offspring to hatch.

Insecticides can have transgenerational effects, affecting offspring hatchability and sex ratios. Thiodicarb significantly reduces the proportion of female *Trichogramma pretiosum* Riley offspring (F1) [48], while chlorantraniliprole impacts the hatchability of *Agrotis ipsilon* (Hufnagel) and *A. segetum* (Denis and Schiffermuller) [49]. In this study, the proportion of females in the offspring (F1) of all three fruit fly species decreased to varying degrees, and the hatching rate of *B. tau* was reduced. This was likely due to a decrease in the quality of eggs after exposure to pesticides, which can affect hatching rates or elicit specific effects on the reproductive cells or developmental stages, influencing sex ratios. These transgenerational effects may involve genetic and non-genetic mechanisms [50,51]. In some cases, pesticide exposure may induce genetic mutations that affect the offspring genome [52]. In other instances, these effects may result from epigenetic changes induced by environmental pressures, which can be transmitted to subsequent generations [53,54]. In our comparison, we found that the differences among the population parameters were not significant. Understanding the transgenerational effects of pesticides is crucial for comprehensively assessing their ecological risks and environmental impacts.

## 5. Conclusions

In this study, we investigated the sensitivity of three fruit fly species to a novel pesticide (fluralaner) and the widely used dinotefuran. Both pesticides exhibited strong toxicity against fruit flies, suggesting their potential for future rotational strategies in field applications. However, the study was confined to laboratory conditions, and specific field applications should consider sublethal effects, as well as the safety of the chemicals to non-target organisms and their environmental impact. In summary, this study on sublethal effects contributes to a more comprehensive understanding of the impact of insecticides on individual insects and populations, as well as their long-term effects on ecosystems. Our findings provide reliable data for the field application of fluralaner and dinotefuran in fruit fly control, as well as for improving IRM strategies.

## Figures and Tables

**Table 1:** Lethal activity of fluralaner and dinotefuran against three adult *Bactrocera* fruit fly species.

Fruit Fly Species	Reagent	Regression Equation	LC_50_ (95% CL; mg/L)	LC_30_ (95% CL; mg/L)	LC_15_ (95% CL; mg/L)	R
*B. dorsalis*	Flu	Y = 1.858x + 0.244	0.739 (0.087–1.336)	0.386 (0.016–1.858)	0.205 (0.003–0.568)	0.899 *
	Din	Y = 2.454x − 1.294	3.366 (2.449–4.472)	2.058 (1.298–2.777)	1.273 (0.659–1.848)	1.000 **
*B. cucurbitae*	Flu	Y = 1.340x + 1.882	0.039 (0.00–0.00)	0.016 (0.00–0.00)	0.007 (0.00–0.00)	0.926 **
	Din	Y = 0.773x − 0.539	4.979 (1.548–16.221)	1.045 (0.004–2.574)	0.228 (0.00–0.978)	1.000 **
*B. tau*	Flu	Y = 0.796x + 0.586	0.184 (0.00–0.940)	0.040 (0.00–0.416)	0.009 (0.00–0.192)	1.000 **
	Din	Y = 0.875x − 0.297	2.183 (0.00–0.00)	0.549 (0.00–0.00)	0.143 (0.00–0.00)	0.899 *

Abbreviations: CL, confidence limit; Din, dinotefuran; Flu, fluralaner; R, correlation coefficient. * *p* < 0.05; ** *p* < 0.01.

**Table 2:** Survival rate, hatching rate, and fecundity of *Bactrocera* fruit flies treated with sublethal concentrations of fluralaner or dinotefuran.

Fruit Fly Species	Reagent	Concentration	Hatching Rate (%)	Fecundity (Eggs/Female)	Survival Rate (%)	
					Female	Male
*B. dorsalis*	Flu	LC_15_	61.20 ± 5.35 ^a^	344.45 ± 5.77 ^b^	19.93 ± 4.11 ^Ab^	25.83 ± 3.50 ^Ab^
		LC_30_	47.78 ± 5.15 ^a^	254.65 ± 8.08 ^c^	28.93 ± 3.34 ^Ab^	15.33 ± 2.66 ^Bb^
		LC_50_	54.08 ± 5.54 ^a^	429.59 ± 28.87 ^a^	20.90 ± 4.16 ^Ab^	12.96 ± 3.23 ^Bb^
		Control	55.87 ± 5.78 ^a^	367.65 ± 17.32 ^ab^	50.91 ± 6.44 ^Aa^	46.71 ± 5.10 ^Aa^
	Din	LC_15_	52.49 ± 3.95 ^a^	344.56 ± 25.40 ^a^	48.15 ± 4.73 ^Aa^	50.00 ± 4.54 ^Aa^
		LC_30_	49.85 ± 4.10 ^a^	161.24 ± 17.32 ^b^	33.33 ± 3.86 ^Ab^	31.05 ± 3.63 ^Ab^
		LC_50_	56.53 ± 5.44 ^a^	140.45 ± 23.09 ^b^	33.08 ± 4.24 ^Ab^	38.63 ± 3.71 ^Aab^
		Control	57.62 ± 4.70 ^a^	278.76 ± 11.55 ^a^	49.23 ± 4.53 ^Aa^	47.60 ± 4.74 ^Aa^
*B. cucurbitae*	Flu	LC_15_	24.65 ± 2.70 ^a^	232.87 ± 28.87 ^a^	38.78 ± 3.89 ^Bb^	51.00 ± 2.58 ^Ac^
		LC_30_	21.23 ± 2.70 ^a^	217.29 ± 11.55 ^a^	40.80 ± 2.93 ^Bb^	62.40 ± 2.60 ^Ab^
		LC_50_	12.63 ± 1.54 ^b^	137.36 ± 21.36 ^a^	32.56 ± 3.77 ^Bb^	35.48 ± 3.61 ^Ad^
		Control	27.78 ± 2.53 ^a^	234.27 ± 35.28 ^a^	72.37 ± 2.40 ^Aa^	74.42 ± 1.80 ^Aa^
	Din	LC_15_	26.26 ± 3.56 ^a^	241.55 ± 23.09 ^a^	17.95 ± 2.15 ^Bc^	30.96 ± 2.36 ^Ab^
		LC_30_	22.51 ± 2.93 ^a^	185.94 ± 25.98 ^a^	27.56 ± 2.57 ^Aab^	27.44 ± 2.78 ^Aab^
		LC_50_	24.73 ± 3.75 ^a^	165.48 ± 9.04 ^a^	21.54 ± 1.99 ^Abc^	21.60 ± 1.48 ^Ac^
		Control	24.31 ± 2.95 ^a^	237.06 ± 21.40 ^a^	32.84 ± 2.89 ^Aa^	40.22 ± 2.91 ^Aa^
*B. tau*	Flu	LC_15_	43.43 ± 2.72 ^b^	423.66 ± 27.56 ^a^	59.03 ± 2.65 ^Ba^	78.30 ± 1.44 ^Aa^
		LC_30_	45.46 ± 2.87 ^b^	535.91 ± 1.28 ^a^	61.73 ± 2.10 ^Ba^	70.94 ± 2.01 ^Ab^
		LC_50_	51.59 ± 3.32 ^ab^	433.78 ± 18.73 ^a^	55.66 ± 3.25 ^Aab^	54.43 ± 2.10 ^Ac^
		Control	56.10 ± 2.41 ^a^	493.47 ± 37.53 ^a^	47.39 ± 2.26 ^Ab^	53.18 ± 2.41 ^Ac^
	Din	LC_15_	38.65 ± 3.58 ^bc^	277.94 ± 34.27 ^a^	49.46 ± 2.21 ^Aab^	51.35 ± 2.48 ^Aa^
		LC_30_	48.14 ± 3.03 ^ab^	370.17 ± 17.56 ^a^	55.71 ± 2.98 ^Aa^	53.21 ± 1.90 ^Aa^
		LC_50_	32.76 ± 3.23 ^c^	423.06 ± 44.46 ^a^	32.37 ± 2.63 ^Ac^	33.24 ± 0.94 ^Ab^
		Control	51.58 ± 2.94 ^a^	355.60 ± 38.26 ^a^	42.80 ± 2.72 ^Abc^	51.12 ± 3.10 ^Aa^

Abbreviations: Din, dinotefuran; DMRT, Duncan’s multiple-range test; Flu, fluralaner; SEM, standard error of the mean. Data (mean ± SEM) followed by the same lowercase letters (superscripts) are not significantly different (within each single species, vertically) according to Duncan’s multiple-range test (DMRT) at the *p* = 0.05 level. Data (means ± SEM) with the same capital letter (superscripts) are not significantly different (comparing survival rates between males and females within the same treatment, horizontally) at the *p* = 0.05 level according to a *t*-test.

**Table 3:** Population parameters of *Bactrocera* fruit flies treated with sublethal concentrations of dinotefuran and fluralaner.

Fruit Fly Species	Reagent	Concentration	Population Parameter			
			R_0_	T	r_m_	λ
*B. dorsalis*	Flu	LC_15_	86.52 ± 3.55 ^b^	29.27 ± 1.76 ^a^	0.15 ± 0.01 ^a^	1.17 ± 0.01 ^a^
		LC_30_	82.85 ± 7.83 ^b^	27.64 ± 1.91 ^a^	0.04 ± 0.01 ^b^	1.05 ± 0.01 ^b^
		LC_50_	73.17 ± 22.04 ^b^	32.14 ± 3.64 ^a^	0.13 ± 0.02 ^a^	1.14 ± 0.02 ^a^
		Control	176.28 ± 12.50 ^a^	28.84 ± 1.78 ^a^	0.18 ± 0.01 ^a^	1.20 ± 0.01 ^a^
	Din	LC_15_	179.82 ± 13.24 ^a^	37.49 ± 2.52 ^a^	0.14 ± 0.01 ^a^	1.15 ± 0.01 ^a^
		LC_30_	44.84 ± 13.86 ^b^	24.28 ± 6.28 ^a^	0.08 ± 0.02 ^a^	1.09 ± 0.03 ^a^
		LC_50_	74.10 ± 18.51 ^b^	28.69 ± 5.20 ^a^	0.15 ± 0.02 ^a^	1.17 ± 0.02 ^a^
		Control	185.78 ± 11.54 ^a^	35.14 ± 5.54 ^a^	0.15 ± 0.01 ^a^	1.16 ± 0.01 ^a^
*B. cucurbitae*	Flu	LC_15_	134.97 ± 19.63 ^a^	41.47 ± 10.55 ^a^	0.12 ± 0.02 ^a^	1.13 ± 0.03 ^a^
		LC_30_	117.63 ± 9.81 ^a^	47.24 ± 5.47 ^a^	0.11 ± 0.02 ^a^	1.11 ± 0.03 ^a^
		LC_50_	103.07 ± 19.05 ^a^	36.22 ± 15.14 ^a^	0.15 ± 0.03 ^a^	1.16 ± 0.04 ^a^
		Control	190.75 ± 25.98 ^a^	60.21 ± 7.08 ^a^	0.09 ± 0.01 ^a^	1.09 ± 0.01 ^a^
	Din	LC_15_	45.52 ± 14.27 ^a^	46.29 ± 5.91 ^a^	0.08 ± 0.01 ^a^	1.08 ± 0.01 ^a^
		LC_30_	59.03 ± 17.26 ^a^	52.19 ± 2.11 ^a^	0.08 ± 0.01 ^a^	1.08 ± 0.01 ^a^
		LC_50_	43.95 ± 6.52 ^a^	46.55 ± 6.06 ^a^	0.08 ± 0.01 ^a^	1.09 ± 0.01 ^a^
		Control	70.31 ± 15.29 ^a^	61.56 ± 4.94 ^a^	0.07 ± 0.01 ^a^	1.07 ± 0.01 ^a^
*B. tau*	Flu	LC_15_	236.72 ± 19.63 ^a^	73.04 ± 2.22 ^ab^	0.02 ± 0.01 ^a^	1.02 ± 0.01 ^a^
		LC_30_	325.47 ± 31.75 ^a^	79.40 ± 1.80 ^a^	0.01 ± 0.01 ^a^	1.01 ± 0.01 ^a^
		LC_50_	251.13 ± 16.00 ^a^	67.89 ± 1.30 ^b^	0.02 ± 0.01 ^a^	1.02 ± 0.01 ^a^
		Control	218.98 ± 24.25 ^a^	72.52 ± 1.37 ^ab^	0.02 ± 0.01 ^a^	1.02 ± 0.01 ^a^
	Din	LC_15_	141.92 ± 20.50 ^a^	68.19 ± 2.66 ^a^	0.07 ± 0.01 ^a^	1.08 ± 0.01 ^a^
		LC_30_	214.18 ± 26.07 ^a^	71.70 ± 4.15 ^a^	0.08 ± 0.01 ^a^	1.08 ± 0.01 ^a^
		LC_50_	148.54 ± 31.84 ^a^	64.76 ± 1.19 ^a^	0.08 ± 0.01 ^a^	1.08 ± 0.01 ^a^
		Control	151.20 ± 25.63 ^a^	64.68 ± 3.93 ^a^	0.08 ± 0.01 ^a^	1.08 ± 0.01 ^a^

Abbreviations: λ, finite rate of increase; Din, dinotefuran; Flu, fluralaner; R_0_, net reproductive rate; r_m_, intrinsic rate of increase; SEM, standard error of the mean; T, interval between each generation. Data (means ± SEM) followed by the same lowercase letters (superscripts) are not significantly different (within each single species) according to Duncan’s multiple-range test (DMRT) at the *p* = 0.05 level.

## Data Availability

The data presented in this study are available on request from the corresponding author.
